# Cardiac dysfunction in the diabetic rat: quantitative evaluation using high resolution magnetic resonance imaging

**DOI:** 10.1186/1475-2840-5-7

**Published:** 2006-04-04

**Authors:** Rajprasad Loganathan, Mehmet Bilgen, Baraa Al-Hafez, Mohammed D Alenezy, Irina V Smirnova

**Affiliations:** 1 Department of Physical Therapy and Rehabilitation Science, University of Kansas Medical Center, 3901 Rainbow Blvd., Kansas City, KS 66160, USA; 2 Hoglund Brain Imaging Center, University of Kansas Medical Center, 3901 Rainbow Blvd., Kansas City, KS 66160, USA; 3 Department of Molecular and Integrative Physiology, University of Kansas Medical Center, 3901 Rainbow Blvd., Kansas City, KS 66160, USA; 4 Department of Physics and Astronomy, University of Kansas, Malott Hall, 1251 Wescoe Hall Dr., Lawrence, KS 66045, USA

## Abstract

**Background:**

Diabetes is a major risk factor for cardiovascular disease. In particular, type 1 diabetes compromises the cardiac function of individuals at a relatively early age due to the protracted course of abnormal glucose homeostasis. The functional abnormalities of diabetic myocardium have been attributed to the pathological changes of diabetic cardiomyopathy.

**Methods:**

In this study, we used high field magnetic resonance imaging (MRI) to evaluate the left ventricular functional characteristics of streptozotocin treated diabetic Sprague-Dawley rats (8 weeks disease duration) in comparison with age/sex matched controls.

**Results:**

Our analyses of EKG gated cardiac MRI scans of the left ventricle showed a 28% decrease in the end-diastolic volume and 10% increase in the end-systolic volume of diabetic hearts compared to controls. Mean stroke volume and ejection fraction in diabetic rats were decreased (48% and 28%, respectively) compared to controls. Further, dV/dt changes were suggestive of phase sensitive differences in left ventricular kinetics across the cardiac cycle between diabetic and control rats.

**Conclusion:**

Thus, the MRI analyses of diabetic left ventricle suggest impairment of diastolic and systolic hemodynamics in this rat model of diabetic cardiomyopathy. Our studies also show that in vivo MRI could be used in the evaluation of cardiac dysfunction in this rat model of type 1 diabetes.

## Background

Diabetic cardiomyopathy (DCM) is characterized by a cascade of myocardial changes that occurs in diabetes mellitus with fibrosis, hypertrophy and microcirculatory abnormalities. These cardiovascular complications compromise cardiac performance ultimately resulting in cardiac failure. A high prevalence of cardiac failure is seen in individuals with diabetic cardiovascular complications, with DCM as one of the key determinants [1]. DCM is marked by diastolic dysfunction early in the disease progression [2-4], with its reported occurrence even in patients with well-controlled diabetes in the absence of clinically detectable cardiac disease [5]. In addition reports also suggest subtle systolic dysfunction later during the course of diabetes that evades detection with echocardiography [2]. Meanwhile it has been suggested that detection of systolic dysfunction might require highly sensitive techniques [3].

Magnetic resonance imaging (MRI) has proven to be a powerful and robust noninvasive imaging modality for structural and function evaluation of the rat heart [6]. However, in vivo cardiac MRI studies using diabetic rat models are very limited. For example, Al-Shafei and colleagues [7,8] performed elaborate MRI studies with a 2 T magnet on streptozotocin- (STZ) diabetic Wistar rats to assess abnormalities of myocardial structure and cardiac cycle events in diabetes.

Understanding the course of pathological events in an appropriate model is the key for developing therapeutic strategies aimed at preventing the heart failure. In order to evaluate the cardiac performance in vivo we used MRI, a robust technique for resolving cardiac functional information and the reference standard for real time three dimensional visualization of myocardial structure [9,10]. In a previous study, we demonstrated the merits of high resolution MRI in visualizing the diabetic heart and characterized the structural properties of non-beating myocardial tissue in the STZ-diabetic Sprague-Dawley rat [11]. As an extension of our previous study, we have characterized the cardiac dysfunction associated with diabetes in this model. In particular, we report quantitative measurements on left ventricular end-diastolic and end-systolic volumes and demonstrate that these parameters are different for STZ-diabetic Sprague-Dawley rats compared to control rats.

## Methods

### Experimental model of type 1 diabetes

All procedures on rats were approved by the University of Kansas Medical Center Institutional Animal Care and Use Committee. Twelve male Sprague-Dawley rats aged 2 months with an initial body mass of approximately 250 g were used for the study. The rats were randomly assigned to control or diabetic groups (n = 6 per group). The rats in the diabetic group were given a single intraperitoneal dose of streptozotocin (65 mg/kg, Sigma, St. Louis, MO) in 10 mM sodium citrate buffer, pH 4.5. The control rats were injected with the same volume of vehicle. Diabetes was confirmed in the former group by measuring the non-fasting plasma glucose levels (≥ 300 mg/dL) two days following the injection. Body mass and plasma glucose levels were recorded once weekly. All rats were given unlimited access to chow and water for the entire duration of study.

### MRI procedures

At the end of 8 weeks of diabetes, MRI scans were performed on rats using a 9.4 T horizontal bore scanner (Varian Inc., Palo Alto, CA) and a 60 mm radio frequency volume coil while the rats were under 1.5% isoflurane anesthesia delivered via a nose cone in a mixture of air and oxygen (60% and 40% respectively). A cardiovascular physiological monitoring system (SA Instruments Inc., New York, NY) was used to monitor electrocardiogram (EKG), respiratory status, and body temperature. The physiological status of the rats was continuously monitored to ensure stable heart and respiratory rates during the imaging session. The rats were positioned in the magnet bore for imaging the left ventricle (LV). After confirmation of position with scout images, EKG gated gradient-echo based sequence was used to acquire cine images of cardiac cycle from a short axis view of the heart over 10 equally incremented intervals (labeled phase 1 through 10) with the following parameters: TR/TE = 25/2.44 ms, number of averages = 1, image matrix = 128 pixels × 128 pixels, field-of-view = 60 mm × 60 mm, frame rate = 10, number of slices = 1, and slice thickness = 2.0 mm. The image acquisition was repeated for a total of six times by moving the slice location to completely encompass the LV cavity from the base to the apex.

### Image analyses

Images were analyzed using the Image J software [12] at 300% precision zoom. For the purpose of graphical representation and discussion, the cardiac cycle was apportioned into ten phases. The blood filled LV appeared hyper-intense on images, thus providing excellent contrast for manually tracing the boundary of LV endocardium. For each LV slice, the slice volume of the particular phase was computed by the product of slice thickness and area of the manually traced blood disc using the pixel to area conversion factor 1pixel/0.22 mm^2^. The volumes from all six slices acquired during the same phase delay were integrated to obtain the volume of LV at the corresponding phase. These computations were repeated for all ten phases of the cardiac cycle. The phases corresponding to the largest and smallest LV volume were chosen to be representative of end-diastole and end-systole, respectively. The difference between the LV end-diastolic and the LV end-systolic volume was expressed as the stroke volume. The ratio of stroke volume to the end-diastolic volume was expressed as the ejection fraction (%).

The LV wall volume was calculated from the phase 1 reconstruction of all six slices. Briefly, the LV wall was manually traced to obtain the pixel count within the region of interest, and the abovementioned pixel to area conversion factor was used to estimate the LV wall area. The wall volume for each slice was obtained from the product of slice thickness and estimated area [11]. The sum of wall volumes from all six slices was expressed as the total LV wall volume.

### Glucometry and gravimetry

Plasma glucose, body mass, and glycated hemoglobin (HbA1_C_) levels were measured at the end of 8 weeks, one day prior to MRI scans. Plasma glucose levels were measured using AccuCheck Active (Roche Diagnostics Co, Indianapolis, IN) meter. HbA1_C _was determined using antibody based A1CNow meter (Metrika Inc, Sunnyvale, CA). After MRI procedures, rats were euthanized with an overdose of sodium pentobarbital. The hearts were excised, washed in cold phosphate buffered saline, blotted, and weighed.

### Statistical analysis

The data were analyzed using SigmaPlot 2000 software. All the values were presented as group means ± SDs. One-sided independent sample Student's t-test was used to assess the difference between group means. The difference between groups was considered significant when P = 0.05.

## Results

### Animal model characteristics

The animal glucometric and gravimetric characteristics measured at the termination of experiment, for both control and diabetic groups, are presented in Table 1. Diabetic rats displayed dramatically elevated plasma glucose level when compared to controls. Glycated hemoglobin increased beyond the level of measurable range (>13 %), confirming long-term uncontrolled hyperglycemia in the diabetic rats. The mean body mass value was significantly decreased in diabetes. The mean heart to body mass ratio was significantly higher in the diabetic group compared to controls (P < 0.05). All these parameters suggested that the rat model used in this study displayed features characteristic of type 1 diabetes.

**Table 1:** Glucometry and gravimetry data obtained at 8 weeks of diabetes

**Rat group (n)**	**Plasma glucose (mg/dL)**	**HbA1c (%)**	**Heart mass (mg)**	**Body mass (g)**	**Heart to body mass ratio (mg/g)**
Control (6)	110 ± 15	4.7 ± 0.2	1,305 ± 80	420 ± 20	3.1 ± 0.1
Diabetic (6)	545 ± 45*	13* ^#^	1,132 ± 81	292 ± 35*	3.9 ± 0.5*

* P < 0.05 when diabetic rat values were compared to controls.

^# ^– since all diabetic rats had HbA1c levels higher than detectable by the method used, we used the highest detectable value (13%) for statistical purposes.

### Left ventricular characteristics

The entire cardiac cycle of all rats was partitioned into 10 equi-duration phases. There was an insignificant (P > 0.05) increase in the mean R-R interval of diabetic rats (242.5 ± 15.0 ms) compared to controls (216.7 ± 28.7 ms).

**Figure 1:**
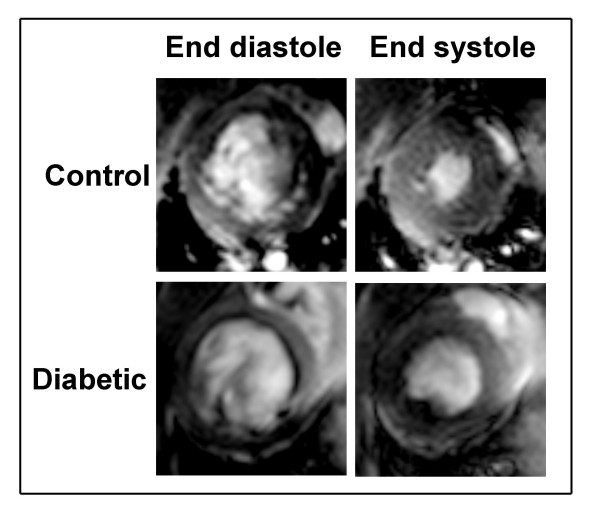
**Representative end-diastolic and end-systolic cine MR images of left ventricle (LV) from control and diabetic rats **Typical slices of LV along the cardiac short axis obtained during end diastole and end systole from age-matched control and diabetic rats (8 weeks diabetes duration) are shown. The blood and the endocardium are clearly distinguished during both phases by the contrast provided by high resolution MRI.

**Figure 2:**
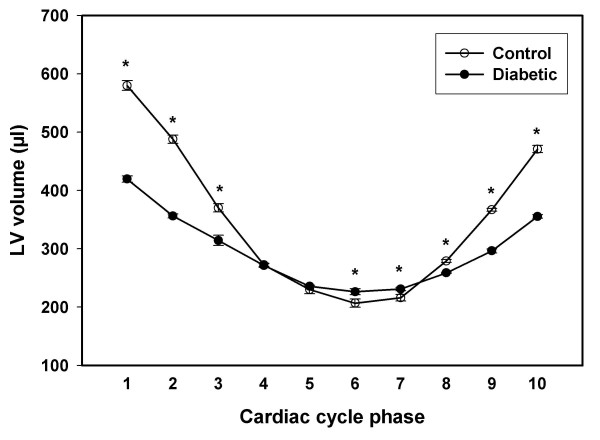
**Left ventricular (LV) volume profiles of control and diabetic rats obtained from MRI reconstruction of LV slices collected throughout the complete cardiac cycle **Graphical representation of LV volumes corresponding to ten equally incremented phases of the rat cardiac cycle is provided. The LV volumes for control (open circles) and diabetic (filled circles) rats were computed from the corresponding MRI scans as described in methods. End-diastole and end-systole correspond to phase 1 and phase 6, respectively, in both the control and diabetic group. LV volumes in all but phases 4 and 5 were significantly different (*, P < 0.05) between groups. Note that the actual cardiac cycle duration was 216.7 ± 28.7 ms in control and 242.5 ± 15.0 ms in diabetic rats, with an insignificant difference (P > 0.05). Hence the cardiac cycle was divided into phases 1 through 10 as discussed in the methods section.

Gating the data acquisition with strong R wave on the EKG signal resulted in the LV attaining maximum volume at phase 1 of the cardiac cycle in both control and diabetic rats. Hence this maximum was taken as the LV end-diastolic volume (Fig. 1). The mean LV end-diastolic volume in the control group was 579.7 ± 8.4 μl, while the diabetic group showed a significantly (P < 0.01) decreased value of 419.4 ± 5.4 μl (Fig. 2).

The LV end-systolic volume was taken as the lowest cardiac cycle phase volume which occurred at phase 6 in both control and diabetic rats (Fig. 1). The mean LV end-systolic volume was 206.7 ± 7.0 μl in the control group and it was significantly (P < 0.01) increased in the diabetic group (226.3 ± 5.3 μl) (Fig. 2).

Subsequently, the mean stroke volume was 373.1 ± 8.8 μl in the control rats. Diabetic rats showed a significantly (P < 0.01) decreased value of 193.2 ± 4.5 μl. The mean ejection fraction remained significantly (P < 0.01) lower in diabetic group compared to the controls (46.1 % vs 64.4 %, respectively).

The body mass normalized mean end-systolic and stroke volumes (Table 2) were significantly (P < 0.01) different between control and diabetic rats, while the normalized end-diastolic volume values demonstrated no difference between groups (P > 0.05). The body mass normalized mean LV wall volume however showed an increase (P < 0.01) with diabetes, suggesting LV hypertrophy in the diabetic rats.

**Table 2:** Left ventricular (LV) characteristics normalized to body mass

**Parameter**	**Control**	**Diabetic**
LV wall volume (mm^3^/g)	1.12 ± 0.30	2.00 ± 0.30*
End-diastolic volume (μl/g)	1.38 ± 0.10	1.46 ± 0.20
End-systolic volume (μl/g)	0.49 ± 0.01	0.79 ± 0.10*
Stroke volume (μl/g)	0.89 ± 0.01	0.67 ± 0.10*

* P < 0.05 when diabetic rat values were compared to controls.

**Figure 3:**
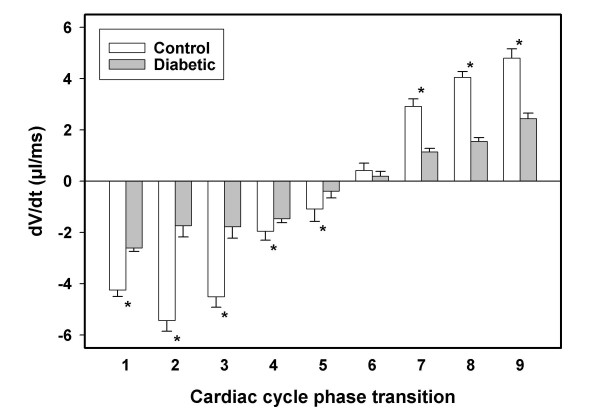
**Cardiac cycle left ventricular (LV) dV/dt values for control and diabetic rats **First derivatives of LV volume with respect to time for control (open bars) and diabetic (filled bars) rats obtained from slopes of secant lines connecting the subsequent phases of cardiac cycle are presented. The x-axis labels refer to phase transitions during the cardiac cycle (for example, '1' corresponds to phase 1–2 transition). The negative dV/dt values correspond to systole and positive values correspond to diastole. The dV/dt values corresponding to all transitions except 6–7 (the end-systolic phase transition) were significantly different between control and diabetic rats (*, P < 0.05).

The first derivatives of the LV volume with respect to time (dV/dt) during the cardiac cycle phase transitions are presented in Figure 3. The dV/dt values remained significantly different (P < 0.05) between the control and diabetic groups at all but the phase 6–7 (the end-systolic phase) transition suggesting a phase sensitive flow velocity difference between control and diabetic LV in this particular model of DCM.

## Discussion

The STZ induced diabetic rats used in our experiments are reminiscent of a model of uncontrolled hyperglycemia due to absolute insulin deficiency. The later feature closely captures the metabolic condition of type 1 diabetes. The STZ rat model has been used to study both tissue pathology [13,14] and therapeutic interventions [15,16] in type 1 diabetes. There has been a growing interest in the application of MRI to obtain structural and functional information from a variety of tissues including the eye [17], the kidney [18] and the heart [7,8,19] that are targeted by diabetic complications.

Functional sensitivity of imaging modalities poses a major challenge for delineation of abnormalities of cardiac function in DCM [3]. However, limitations on functional sensitivity might be lowered with the use of robust non-invasive techniques such as MRI. MRI has evolved as a powerful tool for the evaluation of cardiac function in both humans and experimental animal models of cardiovascular pathology [6,20]. Hence MRI can be applied to the study of cardiac structure and function in DCM. In particular, the MRI study of cardiac abnormalities in DCM provides unique insights into cardiac dynamics that may remain undetected otherwise, with the use of other techniques. For example, echocardiography fails to capture the real state of the tissue due to intrinsic assumptions of tissue geometry [21]. Our gravimetric finding of higher heart to body mass ratio in the diabetic group when compared to the control (Table 1) is suggestive of cardiac hypertrophy and altered ventricular geometry in this rat model at 8 weeks of diabetes. The LV wall volume, calculated from MR images of diabetic rats was not significantly different from that of controls. However with body mass normalization, the mean LV wall volume of the diabetic group became significantly higher than the control group. This supports our gravimetric results and indicates LV hypertrophy in this model of DCM, and is in agreement with our earlier findings reported on non-beating diabetic hearts [11].

In this study we utilized EKG gating to correlate the image acquisition with electromechanical end diastole to obtain functional information on the diabetic LV. The use of cine MRI to image the LV along the cardiac short axis provided excellent temporal resolution to delineate volume changes. The high contrast between the blood and endocardium allowed us to perform the planimetry on LV cavities from all images representing the ten phases of cardiac cycle. LV volume calculations showed a significant reduction of 28% in the mean end-diastolic volume of the diabetic group compared to controls. It has been suggested that the reduction of end-diastolic volume might be the undesirable consequence of an adaptive mechanism of stiff myocardium, in an effort to compensate for poor contractility by increased pressure during experimental cardiomyopathy [22]. A stiff myocardium is characteristic of STZ induced diabetes of similar duration [11]. However the difference in mean end-diastolic volume between groups disappeared when normalized for their body mass suggesting that the role of abovementioned early diastolic adaptive mechanism is plausible in DCM. Meanwhile the end-systolic volume of the diabetic rats increased 10% compared to controls. This difference between groups was also present after body mass normalization suggesting systolic volume dysfunction in this model. As a consequence of disparity between control and diabetic rats in phase volumes, the stroke volume and ejection fraction declined (48% and 28%, respectively) in the diabetic group compared to controls.

The LV end-diastolic volume, stroke volume and ejection fraction displayed significant changes with diabetes in this study, in accordance with a previous report [8]. However, in contrast to our finding of an increase in LV end-systolic volume with diabetes (8 weeks diabetes duration), the previous study (9 weeks diabetes duration) observed no change in this parameter [8]. In addition, the difference in body mass normalized end-diastolic volume between groups was insignificant in our study. Meanwhile recent MRI analyses of cardiac function in 8 weeks STZ-diabetic Wistar-Kyoto rats showed no significant difference in the LV end-diastolic volume, end-systolic volume, stroke volume and ejection fraction from age matched controls [23]. These results may reflect the difference in the strain of rats, since this factor has been shown to clearly influence DCM in the STZ model of type 1 diabetes [24]. Strain differences exist in their susceptibility to DCM with STZ induced diabetes in rodent models [24,25] even though the diabetic cardiovascular complications closely imitate the human condition [26]. In addition, echocardiographic differences in performance have been detected in the two widely used diabetic rat models, viz. STZ-diabetic Wistar [27] and STZ-diabetic Sprague-Dawley [28] rats. The differences in DCM susceptibility and cardiac performance may underlie the manifestation of cardiac functional abnormalities in these models of type 1 diabetes. Meanwhile stroke volume and ejection fraction were decreased in the STZ-diabetic Sprague-Dawley model used in our study, a finding in agreement with results from the STZ-diabetic Wistar model [8], suggesting that the overall cardiac performance is compromised in both models of type 1 diabetes. Interestingly, in a rat model of type 2 diabetes with DCM, the LV end-diastolic volume remained comparable to the age-matched controls while the end-systolic volume was increased due to poor longitudinal contractility of LV [19]. In addition to compromised myocardial contractility in diabetes, the hemodynamic consequences of increased vascular resistance and compromised isoflurane induced vessel wall relaxation may also affect the cardiac cycle systole in these diabetic models [23,29].

The derivatives of volume with respect to time (dV/dt) of the end-diastole to systolic transition and end-systole to diastolic transition were also significantly different between the diabetics and controls, substantiating the pathological changes involving both active (myocytes) and passive (matrix) components, respectively, in the dysfunction of diabetic LV [2,3]. The complex pathology of DCM that limits normal ventricular function involves both cardiomyocyte loss [30,31] and interstitial collagen accumulation [11,16,32]. The loss of force-producing myocytes may underlie contractile dysfunction whereas the accumulation of interstitial collagen might produce difficulties with passive stretch of myocardium during diastole thereby compromising ventricular relaxation in DCM [31,33]. Accordingly, we speculate that a compromised systolic function in this rat model, suggesting loss of contractility may have been the result of myocyte loss due to apoptosis/necrosis that characterize the middle stage of human DCM [3]. Cardiomyocytes demonstrate both impaired contractility and delayed relaxation in mice models of DCM as well [34,35]. Interestingly, the flow velocity of the diabetic group (dV/dt) in this study was not different from the control group at the 6–7 phase transition. This indifference in dV/dt at the end-systolic phase transition suggests that the compromised compliance of the diabetic myocardium is not global, encompassing the entire cardiac cycle. This unexpected result also demonstrates the ability of MRI to provide unique insights that may fail detection otherwise by methods both invasive and non-invasive. However the insignificant difference in dV/dt between the diabetic and control end-systolic phase transition requires cautious interpretation since dV/dt measures are restricted as an indirect index of flow velocity only under assumptions of linear relationship between the variables concerned.

Although our choice of division of the cardiac cycle into equi-duration phases in this study was arbitrary, thus facilitating the evaluation of LV volume with respect to time as a perfectly smooth function between phases, our results nevertheless agree to a substantial degree, with previous reports on normal and diabetic left ventricular function utilizing a slightly different methodology [8,29].

### Limitations of the study

In this study we did not investigate right ventricular dynamics in diabetes. However convincing evidence suggests the impairment of right ventricular function as early as at 6 weeks of diabetes [8]. In addition it may be noted that the LV planimetry in our study was accomplished manually which limits quantitative accuracy. The later limitation could be overcome however in future studies by tailoring software suitable for cardiac functional evaluation. Further, isoflurane has been reported to enhance the ejection fraction of rat hearts [23,29]. The later needs to be taken into account during quantitative cardiac evaluation. However, our study utilized identical anesthetic regimen for both control and diabetic animals to overcome this limitation on the ejection fraction. Finally, in this study we used a single time point of diabetes (8 weeks), although cardiac dysfunction was manifested at this duration of diabetes. Longitudinal investigations will be needed to further characterize the progression of DCM in order to search for effective interventions.

## Conclusion

In conclusion, the results from our investigations indicate that the functional manifestation of DCM in the STZ rat model of subchronic diabetes include early diastolic flow adaptation, systolic volume dysfunction and cardiac cycle phase dependent diminution of LV kinetics. Our study also demonstrates that in vivo MRI is capable of evaluating the cardiac dysfunction in this model of diabetes.

## Abbreviations

DCM – Diabetic cardiomyopathy

LV – Left ventricle

MRI – Magnetic resonance imaging

STZ- Streptozotocin

## Competing interests

The author(s) declare that they have no competing interests.

## Authors' contributions

RL carried out the diabetes induction procedures and rat maintenance, helped with data analysis, statistical procedures and drafting the manuscript. MB conceived of the MRI methods and helped with drafting the manuscript. BAH carried out the MRI procedures. MDA helped with data analysis and statistical procedures. IVS conceived of the study, participated in its design and coordination and helped to draft the manuscript. All authors read and approved the final manuscript.
